# Gathering policymakers’ perspectives as an essential step in planning and implementing palliative care services at a national level: an example from a resource-limited country

**DOI:** 10.1186/s12904-022-00936-1

**Published:** 2022-03-31

**Authors:** Hammoda Abu-Odah, Alex Molassiotis, Justina Yat Wa Liu

**Affiliations:** School of Nursing, The Hong Kong Polytechnic University, Hung Hom, Hong Kong

**Keywords:** Resource-limited countries, National level, Palestine, Palliative care provision, Policymakers, Qualitative study

## Abstract

**Background:**

Despite increasing recognition of the role played by palliative care (PC) services, the priorities of policymakers in supporting PC remain unclear and have sometimes engendered controversy. There are few studies exploring policymakers’ perspectives towards PC services, with most shedding light on obstacles to PC development. Furthermore, no study has explored policymakers’ perspectives towards providing PC at the national level in resource-limited countries. This study provides a platform for providing PC as part of the Palestinian healthcare system (HCS) by exploring policymakers’ perspectives on PC, an essential step to developing a PC programme.

**Methods:**

A descriptive qualitative study design was employed using semi-structured interviews. Participants were those identified as responsible for making executive and legislative decisions about health services (including PC) in the HCS. Data were analysed using qualitative content analysis.

**Results:**

Twelve decision and policymakers participated in the study. Four categories were generated from the content analysis: (1) the nature of current PC healthcare services, (2) the potential benefits of PC, (3) challenges to providing PC, and (4) considerations in providing PC. The current PC services provided to Palestinian patients with life-limiting illnesses and their families are not comprehensive, and are limited to symptom management. There is a Palestinian national strategic plan for developing PC; however, the development goals are not clearly defined, and the plan’s capabilities are inadequate. Several challenges to the provision of PC were found to relate to issues of education and training, the allocation of funding, and the availability of medications.

**Conclusions:**

Integrating PC into the Palestinian university curricula as a compulsory course and establishing higher degree programmes in PC to overcome the shortage of PC specialists is required. Developing policies aligned with national laws could help enhance health services to patients and their families and resolve several challenges. Cooperating with national and international institutions in seeking funding could boost PC development and medication availability.

**Supplementary information:**

The online version contains supplementary material available at 10.1186/s12904-022-00936-1.

## Background

Palliative care (PC) has been highlighted as a top national priority by governments worldwide [1]. It is ranked as the top policy priority in low- and middle-income countries (LMICs) [2]. There are many reasons for this, one being that it is estimated that deaths from a disease indicating PC are projected to increase from the current 25% to more than 42% by 2040 because of rapidly ageing populations [3]. The incidence and prevalence of non-communicable diseases are also rising worldwide due to demographic changes [4]. Considering these facts, many countries have been working towards developing PC by setting PC policies based on their resources and the goals of their healthcare system (HCS). Other countries are updating their policies to ensure that PC services are accessible to all patients. However, a number of countries are still struggling to introduce PC services in their HCS [5], particularly in LMICs.

There are still many challenges hindering the introduction of PC services into HCS in LMICs [6]. One-third of the challenges fall within the policy aspect [7]. A lack of support from policymakers and of a comprehensive national plan for implementing PC are the main challenges to PC provision [8]. The engagement of policymakers in developing PC is critical, as they have a pivotal role to play in developing such programmes at the national level. Therefore, information on the views of policymakers is needed before providing PC; other stakeholders’ views (healthcare professionals (HCPs), the general public, and patients) are also needed to obtain a complete picture of the situation [9]. Involving and exploring policymakers’ perspectives early in the stage of PC planning and development is a significant strength as this can help in the task of identifying issues/policies on which more focus should be placed. Policymakers are responsible for making important decisions related to financial, governance, and service delivery arrangements. Discussing policy-related issues is critical for the future direction of the development of PC services and will help in setting priorities for the provision of PC.

Notwithstanding the increasing recognition of the role played by PC services, the priorities of policymakers in supporting PC are still unclear and have sometimes engendered controversy [1]. Policymakers can undertake several actions to facilitate the the provision of PC programmes, including developing policies, guidelines, and strategies for implementing decision-making processes that support the provision of PC services [5]. Introducing PC within a country’s HCS requires prioritization by policymakers; policies, plans/strategies and guidelines for measuring and supporting progress in PC; and effectiveness in provisioning across settings [1, 10].

Few studies have been carried out to explore the perspectives of policymakers towards the provision of PC services, with most shedding light on obstacles to PC development [1, 11, 12]. Furthermore, most of the evidence is focused on the micro level (daily patient management) [13], with less attention paid to the macro level (roles and responsibilities of decision-makers and policymakers) [1, 11]. Ansari’s study [11], which focused on the factors affecting PC policymaking from the perspective of Iranian stakeholders, showed that the lack of clinical guidelines on PC and financing were the main factors affecting the design of PC systems. Sleeman’s qualitative documentary analysis study [1] shed light on the extent to which PC has been prioritized by the UK government. The results showed that half of the national strategic plans for overall health and wellbeing did not mention PC; only 4% of strategies included PC as a priority area. Despite the findings of previous studies, which provided guidance to countries in developing a PC plan, each country has a unique position and different views of PC as a part of their HCS [14]. This study focused more on PC policy-related issues than did previous studies. Moreover, no study has yet explored the perspectives of policymakers towards providing PC at the national level in resource-limited countries.

This study was conducted in Palestine’s Gaza Strip (GS), where finances are constrained and resources limited [15]. It differs from other countries in the areas of wealth, health insurance coverage, health expenditure, availability of treatment, quality of services, workforce, culture, and religion. Primary healthcare services in the GS rank as the best among neighbouring Arab countries; however, tertiary healthcare services need improvement [16]. Healthcare services, such as PC, targeted cancer therapies, and bone-marrow transplants are limited or fragmented in the GS and require crucial support from policymakers in Palestine. [16]. There is a need for PC in the GS due to demographic changes in population growth and to the increasing number of patients with chronic diseases, particularly cancer, diabetes, and hypertension [17]. To provide a suitable platform for the provision of PC as part of the Palestinian HCSs, there is a need to comprehensively explore policy-related issues from the perspectives of policymakers in the context of the existing HCS [18]. The specific objectives of this study were: (1) to understand the extent to which PC has been identified as a priority by policymakers, (2) to discuss with policymakers existing and new policies that support the integration of PC into the structure of the national HCS, (3) to explore policymakers’ perspectives on the policies and work that have been carried out on PC training and education (4) to identify which essential medicines for pain and symptom management are available in the HCS, their cost, and prescription-related issues from the point of view of policymakers, and (5) to identify the challenges and facilitators to the provision of PC from the perspective of policymakers.

## Methods and analysis

### Study design

An exploratory-descriptive qualitative design was employed in this study. Semi-structured interviews were used to explore the policymakers’ perspectives on PC provision. This study has been reported following the Consolidated Criteria for Reporting Qualitative Research (COREQ) guidelines [19].

### Participants and setting

The participants were those identified as having a policymaking role in the HCS, who were responsible for making executive and legislative decisions about matters related to health services (including PC). These policymakers were from different ministries and held diverse positions, ranging from Deputy Minister of Health, who represents the top level of the Ministry of Health (MOH), to individuals involved in making decisions and writing health policies and guidelines, to healthcare experts working with cancer patients, to representatives of health-related disciplines such as the Palestinian Medical and Nursing Council. Healthcare experts (head nurses and physicians) in the current study are those who have clinical roles in caring for patients with cancer and who have leadership or decision-making responsibilities. They were asked about the needs of cancer patients and the services provided to them. Participants, such as the Director of Doctors Affairs in the Hospital Administration, the Deputy General Director of Nursing, Hospital Managers, and the Head of the Nursing Policy and Quality Department, have strong political, managerial, and decision-making power in planning and putting forward policies for the government to consider.

### Recruitment of participants

A purposeful sampling (criterion-inclusion) approach was utilized in this study, as the aim was to identify and select participants with rich information related to the phenomenon (PC) of interest. Based on this criterion, policymakers who play a role in the provision of care for patients with cancer and who have leadership or decision-making responsibilities, were invited to participate in this study. A preliminary list of potential participants was prepared based on the first author’s knowledge of the literature and on his experience with the local (Palestine) HCS, having worked for more than ten years in three healthcare sectors in Palestine: the clinical field, the General Directorate of Nursing, and higher education (universities).

The first author (HAO) contacted the office of all potential participants via telephone and e-mail, requesting a meeting to explain the study. An invitation letter was then prepared to describe the purpose of the study, along with a consent form and the interview guide, all of which were sent to the potential participants. For participants who responded positively, an interview was arranged at a time and place convenient to him/her. A reminder invitation was sent to those who did not respond to the first invitation within two weeks. If there was no response after two attempts at extending an invitation, no further contact was initiated.

To gain sufficient data for a detailed description of the phenomenon and an in-depth exploration of the topic, 12 participants were required. The sampling approach used in this study was not typical of qualitative studies that depend on data saturation [20], as the study participants were a small, well-defined group of individuals in leading political or management positions. Data saturation was less applicable or appropriate in this study [21]. The number of participants in this study represents the different specialities that play a role in developing health services in the GS. That number could have increased by asking the interview participants to nominate other participants. Each policymaker was a representative of the institution directly linked to the phenomenon/area under study. The goal was not to achieve data saturation but to obtain data from all available key policymakers. Of the 12 participants who were invited, 11 agreed to participate. One person in a key ministerial position forwarded the invitation to the medical director of the ministry due to time constraints.

### Data collection

All of the interviews were conducted in Arabic by the first author, a male PhD candidate from the GS in Palestine, who has previous experience in qualitative research. An interview guide was developed based on previous literature [6, 12] and the authors’ experiences. Two national and three international scholars who are experts in PC policies commented on the guide (supplemental Table S1). The guide was pretested with two head nurses and two nurses with a PhD in nursing and experience in conducting qualitative research. The pretest data were not added to the final data set. Interviews were conducted face to face at the participants’ workplace. All interviews were audio-recorded in the participants’ native language of Arabic and transcribed verbatim. Each interview lasted from 45 to 60 min. The participants were given both Arabic and English versions of their transcript to comment upon and provide feedback on accuracy. After receiving their comments and feedback, the transcripts were integrated into the analysis. Field notes were taken during and immediately after the interview and were cited in the verbatim quotes.

### Data analysis

A qualitative content analysis approach was adopted to analyse the interview data through five steps [22], namely: (1) Audio-recording all interviews in the participants’ native language of Arabic and transcribing them verbatim within the same day, listening to the audiotapes several times to ensure the accuracy of the verbal transcriptions and to become immersed in the data. (2) Selecting meaning units, which refers to the constellation of the words or sentences that refer to different aspects of the policymakers’ perspectives towards PC, using an inductive approach. (3) Labelling every meaning unit with a code. (4) Grouping together subcategories of codes by comparing similarities and differences between them. (5) Developing categories. The first author inductively coded the first three interviews, and all of the team members agreed on the codes/categories after discussions. The subsequent interviews were analysed using both inductive and deductive approaches. MAXQDA 10 facilitated the management of the data.

### Rigour

The Guba and Lincolin [23] method was adopted to ensure the trustworthiness of the study. Credibility was ensured by a peer debriefing on coding conducted by the research assistant, who is a male with a PhD in Public Health and experience in conducting qualitative research. A detailed audit trail of how the data were coded and of the changes made to the codes was created in MAXQDA. Revisions made to the data, audit trail, and participant observations were also reviewed and discussed continuously with the first author and research assistant to confirm the accuracy of the categories/codes that were identified. The Arabic transcripts were sent to the research assistant for coding. The first author and research assistant met to discuss the codes that were generated from the data and to compare similarities and differences between the codes. Similarities were reported in almost all of the codes. Differences were only reported in the choice of some words (linguistically), which were adjusted/modified afterwards. Confirmability was assured by having the other team members review the data and the audit trail in order to bring transparency to the whole study process. A detailed description was given of the setting, participants, and raw data, and of the researcher’s interpretations, to allow readers to judge the transferability of the study.

## Findings

### Characteristics of the participants

A total of 12 decision-makers and policymakers from different organizations (10 from the MOH, one from the Palestinian Medical Council, and one from the Palestinian Nursing Council) participated in the study. The participants from the MOH represented different directorates/sectors: pharmacy, nursing, medicine, policy and quality improvement, and legislative. Most participants were male and held postgraduate degrees. Eight participants had more than 20 years of experience at the MOH.

### Qualitative results: categories and subcategories

The qualitative interviews generated four primary categories of content and eleven subcategories, as shown in Table 1.

**Table 1:** Summary of categories and subcategories of the policymakers

Subcategory	Exemplar comments from participants
**Category (1): Nature of current PC healthcare services**	
• Lack of recognition for holistic PC services	*“…the psychological aspect is the aspect that has not received the required focus. If we want to say it is ignored, some people may be upset, as they are not completely ignored, but rather in attempts, but they are not within a specific template or protocol. They are semi-autonomous activities, but we hope, through the work of the mental health committees, that there will be clear protocols and programs for oncology patients”. (P2, Male, Deputy GD)* *“Most of the care provided to patients with chronic diseases is medical treatment and follow-up in outpatient clinics. About cancer, they are not different from the rest of the chronic patients, although they have a special peculiarity as they are the most patients who need psychological, social and other supports” (P7, Female, Head Clinical Department)* *“…some of the services provided to oncology patients are insufficient, as most of the services focus on physical services. Some psychological, social, and nutritional support services are provided to cancer patients, but they are not in a systematic manner”. (P11, Male, Nursing Consultant)*
• The subordinate status of PC to acute care	*“To be honest, in the MOH strategic plan 2021–2025, PC is mentioned as one of the priorities”. (P12, Male, Medical Director)* *“This is a difficult and embarrassing question, specifically for the MOH, because sometimes the priorities fluctuate. If we talk about the past year, PC was among the main plans of the Ministry. Still, the Corona pandemic changes the priorities and focuses on Coronavirus… Perhaps in the period before Corona, the Ministry’s concerns were focused on emergencies due to the wars. Sometimes some priorities could advance, while others might be delayed based on the unstable conditions in the GS”. (P2, Male, Deputy GD)* *“I cannot answer accurately whether PC is among the Ministry’s priority or not, but I mean they have formed a PC committee, and I am a member of it. I believe that the Ministry has placed PC within the Ministry’s strategic plan, but I do not know to what extent. Putting PC into the plan is one thing and putting the capabilities to implement the plan is another thing”. (P4, Male, Representative Palestinian Medical Council)* *“To be honest, in the MOH strategic plan 2021–2025, PC is mentioned as one of the priorities. There is a timetable for providing PC within the health system, and it is over the next five years. The MOH plans to have PC in every hospital in Gaza. Uh, all this is in the plan, but nothing is happening yet”. (P12, Male, Medical Director)* *“….PC is not among the MOH priorities. All cancer patients are not among the priorities of the Ministry. You are talking about patients who suffer from chronic diseases throughout their lives, and the Ministry does not care about them. MOH focuses on saving cases where there is hope for recovery, such as a case under operations. I mean, the disease is treated, but MOH deals with cancer patients as if they are dead”. (P9, Female, Deputy Head Pharmaceutical Department)*
• Healthcare policies and practice that ignore family needs for PC	*“….We, as a health staff, strive to provide the families with the information they request, as most of them ask about disease progress and the length of time for the patient to live, and we try as much as possible to give them information and support them psychologically…. There is no special place where we provide information. We provide information in the corridor or on the counter”. (P8, Male, Head Clinical Nursing Department)* *“Of course, there are no services provided to the patient’s family. The reason may be due to the lack of interest from the Ministry, and the political situation may also play a role, as most patients are referred to receive service outside the GS. In addition to the pressure on a health system that may play a role in the incompleteness of health services”. (P1, Female, Head Policy Department)*
**Category (2): Potential benefits of PC**	
• Healthcare system benefits	*“PC is critical to the GS…Introducing PC into Gaza hospitals will enhance the quality of services provided as well as will decrease hospital readmission.” (P4, Male, Representative Palestinian Medical Council)*
• Patients’ benefits	*“…by God, in general, the entire GS needs PC (hahaha). We all have chronic diseases. However, cancer patients and patients with chronic diseases in the first place are the most people in need of comprehensive and complete PC because we are talking about a lifelong concern that is not a passing issue like an acute appendicitis patient”. (P2, Male, Deputy GD)*
**Category (3): Challenges to PC provision**	
• Lack of political and social infrastructure for PC	*“Although there are no PC policies in the GS, which is one of the obstacles, there is no problem in developing policies and adopting the policies of neighbouring countries that are similar to us in the system and culture”. (P3, Male, Nursing supervisor- Shifa Hosp.)* *“…The lack of policies is one of the major challenges in introducing PC into the Gaza HCS. We do not have national policies for PC. Perhaps because this is a new issue in the country”. (P12, Male, Medical Director)* *“…We don’t have a specific budget. All budget-related issues of the MOH are managed centrally with the Ministry of Finance… the MOH does not have its own independent budget for development; its budget is at the Ministry of Finance… As I told you, we do not have a special budget to develop the basic services of the MOH, such as purchasing equipment and others, Not to talk about PC…To allocate a budget for PC, we need to have a special budget, and this is difficult in the GS because this is an old system, which is that all ministries’ revenues are directed to the Ministry of Finance, and the latter is the one that pays salaries, development, and others.” (P12, Male, Medical Director)* *“…No, no, no one represents us in the Ministry of Finance. There is no special budget for the MOH, will they develop PC. There was only a budget for the Norwegian project “Pain Management” because it was from external funding, so it had a budget. I mean, if the donor did not support the project, it would not have been accomplished”. (P12, Male, Medical Director)*
• Limited human resources in PC	*“Unpreparedness of HCPs in terms of knowledge and training is one of our problems. The training we are currently carrying out is based on the personal diligence of the committees and YouTube follow-up. No experts are preparing the teaching and training material, which is also one of the obstacles”. (P3, Male, clinical nurse supervisor)* *“A major problem is that we have an increase in the number of cases, but unfortunately, there is no increase in the number of medical staff, including specialized doctors and nurses”. (P1, Female, Head Policy Department)* *“…The most important issue is that we are trained by ourselves, and from my point of view, this training is still insufficient. We do not have staff in Gaza who have trained abroad or have fellowship in PC”. (P7, Female, Head Clinical Medical Department)* *“We don’t fully understand the concept of PC. PC may mean that the patient lives the remaining days of his life without pain. Most of our focus is on pain medication such as morphine”. (P2, Male, Deputy GD)* *“….policymakers are not aware of the importance and benefits of PC. I think it is important for people who have experience in this field to knock on the doors and talk with decision-makers about the importance of PC”. (P1, Female, Head Policy Department)* *“…I prefer to attract experts who speak Arabic because a number of health teams find it difficult to understand English. We know medical terms, but there are words we don’t know. For example, my English is weak, and this honestly did not make me benefit a lot from the course because I could not understand the whole course”. (P7, Female, Head Clinical Medical Department)*
• Unavailability of essential Medication	*“Of course, we, as the Palestinian MOH, adopt the WHO essential drug list. Unfortunately, many medicines are not available for several months, and this is a problem that affects the lives of patients…Continuous interruption of narcotic drugs is among the problems. The interruption and lack of medicines can last for a month or two, and patients suffer a lot from that…. this requires families to buy them…The stores of the MOH in Gaza do not contain medicines. I mean, those here in Gaza don’t want to buy medicines from their budget and wait for the MOH in Ramallah to send them medicine. Here the patient is in a big problem between the two ministries and the conflict between them…For instance, the medicine of Bicalutamide, that the patient takes them at home - of course not available in the Ministry… There are medicines that you find available this month and the next month will not be available…. The interruption and lack of medicines can last for a month or two, and patients suffer a lot from that.” (P9, Female, Deputy Head Pharmaceutical Department)* *“Some doctors write a prescription for the patient so that they can buy it from external pharmacies, but unfortunately, some pharmacies say that they do not have it, they hide it and they sell it to addicted people at a higher price. It means the patient is lost. Or if the outside pharmacist wants to sell the Tramadol, he sells it at a high price for the patient. I mean the addicts in Gaza ruined the patients. I mean, some pharmacies blackmail patients and sell them treatment at a high price” (P9, Female, Deputy Head Pharmaceutical Department)* *“…we are forced to refer a patient to Ramallah for treatment due to the unavailability of medicine in Gaza. I mean, if treatment were available, we would spare the patient the suffering of travel. Some patients travel only to receive a dose of medicine, although we have the capabilities in Gaza to give doses, but Not available”. (P12, Male, Medical Director)* *“…we just want money to solve the problem of the medicine. I mean, if the dispute between Gaza and Ramallah is resolved, the whole problem will be resolved. I mean, most of the medicines are cut off in Gaza are available in Ramallah. Because of the conflict and division, we have two ministries, one in Gaza and the other in Ramallah, and the people and patients are lost among them. I mean, hospitals in the West Bank, the health service situation in them is excellent, and all medicines are available, but Ramallah punishes Gaza, so some medicines are prevented from them, but if there is reconciliation, I think things will improve and medicines will be available”. (P12, Male, Medical Director)*
**Category (4): Considerations for PC**	
• Beginning PC in a centralised location	*“The GS being small, I believe that we need PC program in major hospitals. For example, the PC unit in major hospitals and three community teams cooperate with these hospitals”. (Male, A representative of Palestinian Medical Council)* *We need the Hospital Approach for the acute cases for the patients hospitalised for one or two days. Then when the patient is discharged, their house will be followed by telephone or visited at home”. (Male, A representative of Palestinian Medical Council)*
• Development of PC Policies	*“…. During the next five years, policies for cancer and PC will be developed and will be reviewed by experts and will train the staff on them”. (P1, Female, Head Policy Department)* *“There are treatment protocols for cancer, and these are drug protocols according to the type of disease and the degree of its progression. Also, chronic diseases have their own protocols. There is no problem in the availability of treatment protocols and policies despite the siege.“ (P2, Male, Deputy GD)*
• Healthcare professional capacity building	*“…In 2011, I began the initiative to spread the culture of PC among physicians because the idea did not exist at all. This is the reason why I did not know anything about PC… From 2011 until 2015, 5 workshops were carried out for medical staff… In 2015, I started expanding this work by including a team from Britain and Scotland who are experts in PC… We formed a Steering Committee for PC, and the goal of the committee was to spread the culture of PC and integrate it into the curricula of medical, nursing and physiotherapy students and present it within the services provided by the Palestinian MOH and the UNRWA”. (P4, Male, Representative Palestinian Medical Council)* *“We recently started training the staff and we focus only on nursing. I think nurses are more familiar with the concept of PC than doctors. Doctors do not read about this aspect compared to nurses in the GS who are interested in PC… Also, in the curriculum, the concept of PC is not comprehensively and adequately addressed”. (P1, Female, Head Policy Department)* *“…We have focused on PC in recent years in hospitals, so the health staff will certainly have limited knowledge. The number of courses that dealt with PC is few, and their duration is very, very short, and they were limited to nursing only and medical students at the Islamic University. Physicians working in hospitals need training that is not targeted in PC courses. Also, we did not focus in our courses on Advanced PC, it was the concept in general”. (P3, Male, clinical nurse supervisor) *

*GD* general directorate, *MOH *Ministry of Health, *PC *palliative care, *P *participants

### Category 1: Nature of current PC healthcare services

This category describes the current PC healthcare services provided in the GS as highlighted by the participants. It is comprised of three subcategories: a lack of recognition for holistic PC services, the subordinate status of PC to acute care, and healthcare policies and practices that ignore family needs for PC.

#### Lack of recognition for holistic PC services

The healthcare services provided to patients with chronic diseases, including cancer do not provide PC in a comprehensive manner, as the participants reported. Most of the participants stressed that although the needs of oncology patients vary with the nature of the disease and of their pain, the services that they receive are no different from those provided to patients with other chronic diseases. Whether for oncology patients or other chronically ill patients, health services are often limited to providing symptomatic management that mainly focuses on curative services. The participants stated that other psychological and spiritual services are available, but are not well integrated into the Gaza HCS.

The participants further noted that the current curative focus of the Gaza HCS does not appear to be commensurate with the needs of patients with cancer and other life-limiting diseases. Generally, the services were insufficient, and where they were available, they were often fragmented, and delivery was a significant area of concern.

#### The subordinate status of PC to acute care

The participants pointed out that the Strategic Plan 2021–2025 clearly stated that PC is at the top of the Ministry’s priorities. This is because of its importance to the GS. In contrast, policymakers working in hospitals noted that PC is not regarded as a priority by the Palestinian MOH, considering the ministry’s significant focus on other services, such as cardiac surgery, maternal and child health, and emergency services where the chances of recovery are high. The participants highlighted the point that even if PC services were a part of the ministry’s strategic plan, there is often a gap between the plan and what happens in practice, as the focus still is on acute care.

#### Healthcare policies and practices that ignore family needs for PC

All of the participants agreed that there were no PC services to support families because of a general lack of interest in implementing such policies. They noted that most of what is presented to families is information about the nature of the patient’s illness. Information about physical and psychological issues, such as the handling of distress and pain, is needed for patients with end-stage cancer. Some participants mentioned the lack of suitable places (specialized private rooms) to provide information or psychological support. Families are being provided with information either in a corridor or near the nursing station.

### Category 2: Potential benefits of holistic PC

This category discusses the potential benefits of providing PC in the Gaza context. The two subcategories are benefits to the HCS and benefits to patients and their families.

#### Benefits to the healthcare system

All of the participants noted that PC services are essential for patients in the GS. They said that PC is particularly important in the GS because the GS has a significant burden of patients with chronic diseases. They thought that if PC were to be fully integrated into the Gaza HCS, this might be a cost-effective approach. In addition, the participants mentioned that integrating PC could reduce patient admissions and pressure on the health system, and may have a positive effect on patient recovery.

##### Benefits to patients

The participants mentioned that all patients in the GS stand to benefit from PC. As an order of priority, they noted that persons with cancer are most in need of this service, followed by those with renal conditions. The participants reiterated that patients in these two categories suffer from such side effects of their disease as psychological pressure and physical exhaustion. Furthermore, patients with advanced-stage diseases have a lower cure rate and often have severe complications, which fit well with PC services.

### Category 3: Challenges to PC provision

This category illustrates several challenges to the provision of PC in the Palestinian HCS. It is comprised of three subcategories: a lack of political and social infrastructure for PC, limited human resources in PC, and the unavailability of essential medicines for pain and symptom management.

#### Lack of political and social infrastructure for PC

The main political and social infrastructure challenges are a lack of policies and a strategic direction on PC, and a lack of funds/budget allocation. The participants attributed the lack of policies to the novelty of this topic in the GS. They expressed the view that the Ministry could adopt PC policies from neighbouring countries with a similar culture, and modify those policies according to the realities of the Palestinian health system.

A lack of funds/budget allocation is another significant challenge to the provision of PC in the GS. The MOH does not have an independent budget. Rather, the Ministry of Finance manages and distributes funds to all of the ministries, including the MOH. The funding problem could be solved by finding donors from abroad to fund the PC programme. There is no MOH representative at the Ministry of Finance. When the MOH needs funds to develop or repair equipment, it sends an official request to the Ministry of Finance and awaits their approval. This takes time, which can lead to significant problems in the meantime for the MOH. The MOH often purchases any equipment or tools from its own funds, which are too meagre to fund and support programmes such as PC.

#### Limited human resources (education and training) in PC

A shortage of specialized/expert PC professionals, insufficient knowledge and training of professionals, misunderstandings and a lack of awareness on the part of decision-makers of the concept of PC, and cultural barriers, including language, are the leading human resource challenges. The participants said that there was a noticeable increase in the number of cancer patients; however, the number of health staff working in oncology departments has not increased. There is little employment compared to the increasing numbers of patients. Providing sufficient numbers of staff trained in PC is a necessary step to take before introducing PC.

The lack of knowledge and training on the part of HCPs is also a significant challenge in the provision of PC in the GS. The policymakers indicated that watching YouTube videos and reading articles are the two methods that professionals in the GS rely on to obtain knowledge about PC. Another challenge is the misunderstanding that decision-makers have of the comprehensive meaning of PC and its importance in enhancing services and reducing hospital admissions. Many policymakers have linked PC with pain management only. Some participants suggested that attempts should be made to convince policymakers of the benefits of PC by talking to them.

Some participants mentioned that there are many workshops conducted in the GS on PC. Most of these workshops are presented by English-speaking experts. This is an obstacle for most attendees. The participants suggested inviting Arabic experts who are knowledgeable about the Arab culture and norms.

#### Unavailability of essential medicines for pain and symptom management

The unavailability of drugs is one of the main challenges to the provision of PC in Gaza. The problem includes a lack of continuity in the availability of drugs and exploitation from private pharmacy owners, despite the MOH having adopted the WHO’s essential drug list.

The lack of essential medicines forces the families of patients to buy them privately, which is a burden on them considering the frequent sieges/conflicts and the high unemployment and poverty rates in the GS. Policymakers attributed the unavailability or interruption of medicines to political quarrels between the MOH in the GS and the MOH in Ramallah, and the lack of funds to purchase medicines, as the Ministry relies on foreign donations. The political situation in the country has affected the development of health services and limited it to acute care services only, not PC, which arguably is an aspect of essential services. The unavailability of chemotherapy drugs forces physicians to refer patients to hospitals in the West Bank for treatment, who would then return to hospitals in the GS to complete their follow-up. This places a psychological, physical, and material burden on patients. Some PC medications are also not available, affecting the lives of patients who suffer from psychological, physical, and other problems.

Extortion by some pharmacy owners is a problem that patients face when seeking narcotic treatments from private pharmacies. These pharmacies will either raise their prices or inform patients that the medications are unavailable, and then sell them to customers who are willing to pay an exorbitant price for them. In an attempt overcome this problem, the MOH, in coordination with some pharmaceutical companies, provides narcotic drugs, such as Tramadol, in hospitals.

When addressing strategies to solve the unavailability or interruption of medicines, the participants indicated that separating health services from political conflict is very important for the sustainability of the delivery of medications. It is also essential to seek donors to be able to purchase the costly medications that are straining the MOH’s budget. Hospitals also purchase medications in case they become unavailable or only intermittently available.

### Category 4: Considerations for integrating PC into the HCS

This category describes the actions needed to integrate PC into the current HCS. It is comprised of three subcategories: beginning PC in a centralized location, development of PC policies, and capacity building (education and training).

#### Beginning PC in a centralized location

 The participants suggested that a hospital-based approach would be the “best [way] to begin PC” within the context of the situation in Gaza. Most patients who have been diagnosed as being in an advanced stage of their disease need to receive treatment in a hospital. Staff shortages in Gaza do not allow the Ministry to adopt any other approach, as noted:

#### Development of PC policies

Policies are statements of intentions that elaborate on the government’s goals and actions with regard to implementing PC services. The participants in this study reported that developing policies in the upcoming five years is the main task of the MOH. There are protocols and guidelines for treating each type of cancer, but policies and protocols for PC are not yet available or have yet to be developed. Protocols, written plans about when and which group can benefit from PC, are more specific than guidelines. Guidelines are statements that have been developed to assist HCPs in making decisions about proper care for a specific patient population, and allow policymakers to monitor progress in the development of PC. A special national committee for PC was formed, and one its tasks included developing policies, guidelines, and protocols for PC treatments.

#### Building the capacity of healthcare professionals (education and training)

Investing in the education and training of HCPs should be considered while developing PC in the GS. Participants emphasized the importance of integrating PC within the curricula and encouraging training in a clinical context. They stated that several attempts had been made since 2011 to disseminate the concept of PC among professionals, particularly physicians and nurses. Within nursing, what is being delivered is courses related to oncology, including a two-hour lecture on PC. These nursing courses came about through the personal diligence of supervisors and heads of oncology departments in hospitals. These courses touched on essential aspects of the WHO model and focused on quality of life (QOL), communication, psychosocial issues, and telling bad news.

## Discussion

This qualitative study adds to the body of knowledge about policymakers’ perspectives on providing PC services within the HCS of a country with limited resources. Currently, the PC healthcare services provided to Palestinian patients with life-threatening illnesses and their families are fragmented in nature. There is a Palestinian national strategic plan for developing PC; however, the development goals are not clearly defined in the plan, and the capacity to implement the plan is inadequate. Several challenges to the provision of PC in Palestine are issues related to education and training, the allocation of funding allocation, and the availability of medications.

Providing optimal end-of-life care to patients influences health outcomes [24]. Most healthcare services provided to Gazan patients are fragmented, and limited to dealing with the symptoms of a disease and the side effects of the treatment. Fragmented healthcare services may negatively affect the treatment regimens and daily living activities of patients [25]. Several studies have underscored a high prevalence of distress and poor QOL among Palestinian patients with life-threatening diseases, including cancer [26, 27]. This might be attributed to the limited services tailor-made to meet the healthcare needs of this specific patient group. Limited and underdeveloped healthcare services have been ascribed to limited resources and the ongoing siege of GS since 2007 [16]. Enhancing end-of-life patient health outcomes is the most cost-effective method for countries with limited resources [28]. The WHO has recommended integrating PC within HCS as a cost-effective mechanism to provide optimal care for patients and their families [25, 29] and to alleviate their distress [25].

Designing a comprehensive national strategic plan is essential for the successful development of PC [30]. There is a Palestinian national strategic plan for developing PC; however, the development goals are not clearly defined, and capabilities to implement the plan are limited. The results of this study are similar to those reported in most low resource countries [31, 32]. There is a need to deploy a sufficient number of HCPs and PC specialists to achieve strategic plans [5]. Malaysia and South Africa, for example, have an adequate number PC specialists; however, the shortage of staff (physicians and nurses) to support those specialists is still a major problem in implementing PC [32, 33]. In the GS, the problem of staff shortages and the lack of PC experts is a complex one [34]. Investing in PC training is a necessary proactive step to take before PC is introduced in the Palestinian HCS.

Several challenges to providing PC in the GS were found in this study. They are similar to those that have been documented in both high-income countries and LMICs. The main challenges identified in the literature are insufficient PC knowledge among HCPs [35] and the lack of a trained PC workforce [6, 35]. In Palestine, the low level of knowledge of HCPs is attributed to the emerging concept of PC in the GS [34]. There is no PC diploma or Master’s degree to train specialist professionals in PC in the GS, nor has PC training been integrated into university curricula. In 2015, PC was only incorporated into the curriculum of the Faculty of Medicine at the Islamic University as an intensive short subject for five days, including hospital training [36]. Accreditation bodies such as the Ministry of Higher Education Accreditation Commission should integrate PC into the Palestinian university educational curricula. Making PC compulsory in courses leading to basic professional qualifications is one of the main strategies advocated by the WHO [37]. Several countries that have met with success in developing PC have found that it is important to transfer and incorporate education in PC within the existing HCS [37]. The insufficient knowledge of HCPs might also be attributed to cultural and language barriers. Because of globalization, it has become easy for organizations to invite international experts to lead workshops [38]. Most of the workshops held for professionals in the GS were presented by English-speaking experts. Not every professional completely understands a lecture delivered in a foreign language. To overcome this obstacle, the responsible bodies in the GS should consider inviting Arab experts to offer training in PC.

Workforce shortages and a lack specialists in PC were commonly identified challenges. There is a significant global shortage of PC experts [6]. Such a shortage may affect the quality of the services provided to patients [39]. Due to financial constraints in the GS, which may prevent the hiring of new staff, the government can make use of volunteers to help overcome the workforce shortage [40]. Utilizing volunteers to care for and provide health and education support is considered an efficient mechanism in LMICs for lowering the economic burden of delivering PC services [41].

Other unique challenges affecting the provision of PC in the GS are misunderstandings on the part of policymakers about the concept of PC, a lack of policies and legislation on PC, limited resources, and the unavailability of narcotic drugs. These challenges are congruent with those reported in LMICs [6]. Palestinian policymakers have restricted their recognition of PC to the scope of mental health, which is not in alignment with the WHO’s definition [25]. To date, no policies on PC have been developed in the GS. The provision of PC in a country’s HCS requires policies to support and measure the progress of the effort [1]. It has been suggested that PC policies are a significant driver of the introduction of optimal care [1]. According to the Atlas of PC in the Eastern Mediterranean Region, Jordan, Lebanon, and Qatar have developed national policies for providing PC services [42]. Sudan has established a national cancer control programme that sheds light on the early detection, prevention, and enhanced treatment of the disease, and on PC [43]. Although Palestine has also designed comprehensive national cancer control programmes, gaps have been reported in the cancer control strategy [44]. These gaps are mainly related to financial constraints, poor administrative coordination, shortages in human healthcare resources, and an inadequate workforce, which affect the development of PC services [45]. The Gaza context plays a role in determining the priorities of the MOH in terms of the services that they provide. Thus, Palestine needs to build its policy development capacity and provide guidance, utilizing national and international policy frameworks to combat these challenges. It is also essential to develop policies that align with national laws and engage policymakers in practical/effective policy dialogues.

For the future provision of PC in the Palestinian HCS, the study participants stated that a hospital-based PC approach is the best one to take. No evidence has been given about which approach best fits the setting [46]. Decisions about what types of approaches are suited are based on a country’s context, resource availability, and the goals of their HCS [47]. However, the WHO strongly recommends integrating PC within hospitals, particularly in countries with a high number of incurable chronically-ill patients [48]. In Palestine, there is a significant number of patients with advanced-stage diseases [49]. The effectiveness of adopting such an approach has been underscored in recent studies, including decreasing lengths of stay [50] and reducing hospital costs [50]. Therefore, a hospital-based approach seems to be the best fit for the Palestinian situation to overcome scarce financial resources, a shortage of staff, and limited infrastructure.

### Strengths and limitations of the study

 Utilizing semi-structured interviews may have prevented the participants from expanding their perspectives on the topic, compared with unstructured interviews. However, our participants had a variety of specialities and leadership roles. At the same time, interviewing well-known decision-makers and policymakers may have prevented them from speaking freely about PC-related issues, such as funding.

## Conclusions

This study reveals that Palestinian decision-makers and policymakers view PC as an important service that should be implemented in the HCS. PC services are still limited because of the significant challenges that obstruct the development of PC in the HCS. Integrating PC into Palestinian university educational curricula as a compulsory course and establishing higher degree programmes in PC to overcome the shortage of PC specialists is required. Developing policies aligned with national laws could help to enhance healthcare services to patients and their families and resolve several challenges. Cooperating with national and international institutions in seeking funding could help in the development of PC and availability of medications.

## Supplementary Information

**Additional file 1.**

## Data Availability

The datasets used and/or analysed during the current study are available from the corresponding author on reasonable request.

## Abbreviations

GD: General Directorate
GS: The Gaza Strip
HCPs: Healthcare professionals
HCS: Healthcare system
LMICs: Low- and middle-income countries
MOH: Ministry of Health
PC: Palliative care
QOL: Quality of life
