# Supplementary material for: Gathering policymakers’ perspectives as an essential step in planning and implementing palliative care services at a national level: an example from a resource-limited country
Source: BMC Palliat Care. 2022 Mar 31;21:43. doi: 10.1186/s12904-022-00936-1 (PMC8967559; doi:10.1186/s12904-022-00936-1)
Supplement: Supplementary file 1 — Additional file 1. [file 12904_2022_936_MOESM1_ESM.docx]

**[Supplemental table S1](https://spcare.bmj.com/content/early/2021/08/08/bmjspcare-2021-002982#DC1): Final set of an audio-recorded interview guide**

| **Study Objectives** | **Questions** | **Probe** | **Type of participants will be offered the question** |
| --- | --- | --- | --- |
| Warm-up enquiries | 1. *Could you briefly describe your position/role? Especially your role in developing health services and policies.* |  | All participants |
|  | 1. When we talk about patients with advanced diseases*, how are they currently managed?* | - Probe about services supports them. - Probe about services available to families. - Probe about existing policies for managing patients with advanced diseases. | All participants |
|  | 1. So, we will now be talking specifically about PC; *what does PC mean in the Gaza context?* | - Probe further to which group of patients’ PC should be provided? Why do you consider this patients group? | All participants |
| 1. To understand the extent to which PC has been identified as a priority from policymakers' perspectives. | 1. *How does the government consider PC as a priority in the current healthcare system?* | - If yes, probe further to what extent/level PC is considered a priority to the Ministry? - Probe further about reasons for PC to be a priority at this level.   -----------------------------------------------------------------------------   - If say no, ask why it is not a priority? | All participants |
|  | 1. *How is the government or hospital planning to introduce PC into the healthcare system of Gaza?* | - If yes, probe about the approach/approaches adopted. Why you chose this approach? - What are the strategies for achieving this? - Probing question about possible timelines for it]   -----------------------------------------------------------------------------   - [if they say no, ask why do they have no plan? - Ask about future plans? Probing question about possible timelines for it] | All participants |
| 2. To explore the policymakers’ perception about policies (guidelines, strategies/plans, standards, resources) that support the integration of PC into the structure of national HCSs. | 1. *Tell me about the work/policy that is being done by the Ministry to support PC provision.* | - Ask if there *is a national policy for PC provision?* - What type of policy is developed (guidelines, strategies/plans, standards)? - If say yes, how this policy was developed? - Who participates in developing this policy? - How often is this policy reviewed?   -----------------------------------------------------------------------------   - If say no, why do they have no plan? - Ask about plans for developing national policy? Probing question about timelines for it] | All participants |
|  | 1. *From your experience and your position, what types of guidelines/ policies are existing to support PC provision?* | - If yes, what these guidelines are talking about? - Probe further if the guidelines are developed based on patients’ holistic needs- biopsychosocial-spiritual circumstances? - Probe further about a group of patients that guidelines focus on - Probe about the type of guidelines (general or specific). - Probe also about the content of guidelines   **Prompt**: how about addressing suffering reducing intervention and illness prognosis.   - Probe if the content of guidelines quantifies each healthcare provider's role and their interaction and timing of patients’ assessment. - Probe about findings of the study (1) almost all cancer patients in Gaza experienced unmet needs, why?   -----------------------------------------------------------------------------   - If no, ask why it is not developed? - Probe about strategies, future plans or recommendations to develop PC guidelines in the near future] | All participants |
|  | 1. *Based on your pervious clinical experiences, how PC patients’ are managed in hospitals?* | - Probe about plans to improve the care pathways of such patients? | GD of Hospitals  GD of Nursing  Head of oncology departments |
|  | 1. From your experience, what *is the government allocated budget/resource/manpower for PC services?* | - If say yes, probe about the budget estimation for PC services development (e.g., beds, infrastructure, healthcare professional education and training, essential medications, etc.)?   **Prompt**: how the budget is utilised?  Probe about the role of donor support to support PC.   - Probe about plan/strategy of the government for seeking external funds from international organisations?   -----------------------------------------------------------------------------   - If no, why do they have no budget, etc.. - Ask if there are future strategies or recommendations to allocate budget for PC services improvement? | Director of doctors affairs in hospital administration |
| 3. To explore the policymakers’ perspective about policies/work been done regarding strengthening human resources, such as training and education. | 1. *What have been done or will do to prepare frontline workers/clinicians/nurses in PC services, particularly education and training?* | - If say yes, ask how the content of the training was developed? Is it based on the assessment of professionals’ educational and training-related aspects? - Probe further is the training developed based on WHO four main components, including pain and symptoms management, psychosocial support and end-of-life care, and supervision. - Is the content also shed light on communication and conversation skills? - Probe about the place where training had been conducted—in-services, for example. - Probe further about the group of professionals who received training. Why this group? - Probe about the findings of the study (2) that reported less than 50% of professionals knew about PC. Why do you think so? How this situation could be changed. - Probe about the challenges with changing this situation.   **-----------------------------------------------------------------------------**   - If say no, why do they have no plan to do so? - Probe further if the Ministry is planning for integrating PC into healthcare training curricula. | Head of Palestine Medical Council  Head of Palestine Nursing Council  GD of Nursing  Member of National committee of Palliative Care |
|  | 3- From your view, how ‘ready’ is the system or government in Gaza to develop PC. | - If ready. Probe about the level of PC provision? - If not ready. Probe about the reasons of that. (go to challenges questions). - Probe about the future plan for providing PC. | All participants |
| 4. To explore the policymakers’ perspective concerning the availability of essential medicine for pain and symptom management. | 1. *To what extent has the Ministry adopted the WHO essential medicines in PC?* | - Probe about the essential medicine availability into healthcare system? - Probe further about medicines for pain relief and for most symptoms associated with advanced illnesses? - Ask about who prescribe medications? Probe further if there is clinical guideline adopted for prescription of medications? - Probe further about challenges or restrictions that are in place to access essential medications?   If there are challenges. Probe further about strategies to ensure the access to medications. | GD of Pharmaceuticals and Medical Supplies |
| 5. To identify the challenges and facilitators to the provision of PC from policymakers' perspectives. | 1. From your perspective, what are the challenges and facilitators regarding the development of PC into the healthcare system? | - If say yes, probe about challenges based on three policies (structure, human resources, and medications). - Probe about the main challenges to the provision of PC. - *For Structure*, probe about challenges in developing policies, guideline, - *For human resources*, probe about the shortage of staff, training, knowledge of the staff - *For medication,* probe about availability of medication, access to medications. - Probe about facilitators based on three policies (structure, human resources, and medications). | All participants |
| Ending of interview | Is there anything else they would like to add or suggest as the next important step in the process to implement PC. | key aspects of PC that should be prioritised (i.e. education, training, access to opioids) | All participants |

GD: general director; MOH: Ministry of Health; PC; palliative care
